# Blood pressure changes during different methods of resistance training in normotensive and stage 1 hypertensive individuals: a repeated measures cross-sectional study

**DOI:** 10.1186/s13102-025-01097-3

**Published:** 2025-03-14

**Authors:** Roman Jurik, Petr Stastny, Dominik Kolinger, Artur Gola, Tomas Vetrovsky

**Affiliations:** 1 Faculty of Physical Education and Sport, Charles University, José Martího 269/31, Prague, 162 52 Czech Republic; 2 The Jerzy Kukuczka Academy of Physical Education and Sport in Katowice, Mikołowska 72A, Katowice, 40-065 Poland

**Keywords:** Acute cardiovascular responses, Hypertension, Resistance training, Blood pressure, Blood pressure changes

## Abstract

**Background:**

Stage 1 hypertension influences acute cardiovascular responses to resistance exercises and post-exercise recovery. We examined whether the order of exercises, particularly in agonist-antagonist pairings, can alter these cardiovascular responses. This study compares systolic and diastolic blood pressure responses during agonist and agonist-antagonist paired sets of upper and lower-body resistance exercises with a load of 75% repetition maximum in individuals with normotension and stage 1 hypertension.

**Methods:**

A cross-sectional study enrolled 47 participants with sedentary jobs, comprising 30 normotensive individuals (47.8 ± 5.9 years, height 174.8 ± 10.2 cm, weight 77.7 ± 15.4 kg, BMI 25.3 ± 3.6 kg/m^2^) and 17 hypertensive individuals (54.3 ± 6.0 years, 177.6 ± 11.3 cm, 89.8 ± 16.4 kg, BMI 28.5 ± 4,5 kg/m^2^). Acute cardiovascular parameters were measured using an arteriograph, a non-invasive device designed to assess vascular stiffness and cardiovascular health, after each set of resistance training.

**Results:**

No significant differences in systolic blood pressure changes were found between the resistance training methods and aerobic exercise when comparing normotensive and hypertensive individuals. However, significant increases in systolic blood pressure were observed during lower-body exercises (11.3–24.7 mmHg for normotensives and 11.7–24.1 mmHg for hypertensives, *p* < 0.05). Hypertensive individuals showed slightly higher increases during lower-body supersets (*p* < 0.05). Regarding diastolic blood pressure, significant decreases were noted during upper-body resistance training for both groups, especially for normotensives (-10.6 to -13.7 mmHg, *p* < 0.05).

**Conclusions:**

Agonist and agonist-antagonist paired set resistance training for both lower and upper-body exercises resulted in similar blood pressure changes in individuals with normotension and stage 1 hypertension. These findings suggest that both methods may have comparable cardiovascular effects across blood pressure.

**Trial registration:**

The study was registered on ClinicalTrials.gov (NCT06047678). Registration date: 31 August 2023.

**Supplementary Information:**

The online version contains supplementary material available at 10.1186/s13102-025-01097-3.

## Introduction

Resistance training (RT), despite its positive effects on muscle strength [1] and overall fitness [2], can present a stress situation for the cardiovascular (CV) system [3, 4]. This CV load can be beneficial if managed appropriately [3]. During RT, heart rate (HR), systolic blood pressure (SBP), and diastolic blood pressure (DBP) increase in proportion to the weight lifted and the number of repetitions performed [5, 6]. This physiological response is essential to understand, as it underpins the importance of RT design, especially for individuals with varying CV health statuses.

The CV response to RT varies significantly between normotensive and hypertensive individuals [7], with hypertensive individuals experiencing more pronounced changes in blood pressure (BP) during and after exercise [8]. According to the American College of Sports Medicine [9], BP for individuals with hypertension should not exceed 220/105 mmHg during exercise to minimize the risk of complications. Parameters such as exercise intensity and volume play a crucial role in BP responses. For example, Moreira et al. [10] demonstrated that increasing intensity leads to higher BP levels, particularly during submaximal: 85–95% of 1 repetition maximum (RM) [11–13] and maximal exertion: > 95% 1RM [13]. MacDougall et al. [13] observed BP values as high as 480/350 mmHg during leg press exercises at 95% of 1RM until momentary failure, while Gotshal et al. [14] found that higher training volume also elevates BP. These terms refer to the maximum number of repetitions that can be performed with a given load before reaching momentary failure. The volume of work performed, whether through unilateral or bilateral exercises, has distinct effects on BP response [15].

Bilateral exercises, due to the larger muscle mass involved, typically lead to higher BP values compared to unilateral exercises [13]. This difference further emphasizes the importance of exercise selection and volume in managing CV responses during RT [16]. The exercises selection is key, as BP increases with the muscle mass engaged [10, 13, 17, 18]. Therefore, understanding how different RT parameters, such as exercise type and order, influence BP is essential for optimizing training and minimizing CV risk.

Traditional approaches for managing BP include choosing lower intensities [13], extending rest periods between sets and exercises [19], or reducing the number of repetitions [20–22] and sets per exercise [23–25]. These approaches can help maintain more stable BP levels throughout the session. Another important parameter is exercise order, as it can reduce local muscle fatigue [26] and prevent excessive BP response [27]. To manage BP effectively during RT, it is also important to understand the effects of different training methods.

The most common method in RT is to combine two consecutive exercises targeting the same muscle group, known as agonist pair sets, which is referred to as traditional agonist (AgPS) [28]. This method is one of the most widely studied in relation to BP changes [29]. Another approach involves pairing two consecutive exercises for muscle groups with opposing functions, called agonist-antagonist paired sets (AgAnPS). This technique allows for shorter rest periods between sets and an increase in load volume [30, 31]. These physiological effects may provide favorable loading conditions for BP management in both normotensive and hypertensive individuals, although they can also lead to higher neuromuscular fatigue [32].

Despite the well-established research on AgPS, the current literature lacks comparisons between AgPS and AgAnPS methods, particularly regarding BP responses during upper- and lower-body exercises in hyperteindividuals will experience significantly higher BP peaks during RT sessions compared to normotensive individuals, and that AgAnPS will result in lower BP peaks during lower-body exercises than AgPS.nsive individuals. Therefore, this study aims to compare the effects of AgPS and AgAnPS RT on BP values in individuals with normotension and stage 1 hypertension. We hypothesize that hypertensive 

## Methods

### Design

This cross-sectional study included three familiarization sessions and five measurement sessions over 5 weeks. The measurement sessions consisted of four RT sessions—AgPS and AgAnPS RT for both upper and lower body—and one aerobic exercise (AE) session as a control. All RT sessions followed a protocol of 10 repetitions at 75% of 1RM for three sets, with 90-second rest intervals and a 3-0-2-1 tempo. These parameters were consistent across all sessions.

Participants were recruited from two private healthcare facilities via phone or in-person approaches by physicians and staff, ensuring similar exercise levels across participants. The study adhered to CONSORT 2010 guidelines for reporting randomized trials [33] and was registered on ClinicalTrials.gov (NCT06047678). Ethics approval was granted by the Faculty of Physical Education and Sport at Charles University (protocol number 242/2018), according to the Declaration of Helsinki 2013.

### Participants

Between April 2022 and August 2023, participants were recruited from two private healthcare facilities through phone calls and in-person approaches by physicians and staff. Stratification was based on BP measurements taken by a cardiologist in a medical setting. Inclusion criteria included ages 40–63 years, normal BP (< 139/89 mmHg) [34] or stage 1 hypertension (140–159/90–99 mmHg) [35], the ability to participate in moderate-intensity RT and AE, physical activity (but not professional athletes), and non-smoking status. For the purposes of study, we included individuals with stage 1 hypertension who were receiving pharmacological treatment as prescribed by their cardiologist, with no modifications to their prescribed treatment regimen.

Exclusion criteria followed Williams et al. [36] and included conditions such as coronary artery disease, heart rhythm disorders, acute myocarditis, Marfan syndrome, diabetes (type 1 or 2), a history of myocardial infarction or stroke, infections (e.g., COVID-19, influenza) [37], grade 3 obesity (BMI ≥ 40.0 kg/m2) [38], high-stage hypertension (grade II-IV) [39] and dizziness during exercise.

**Fig. 1:**
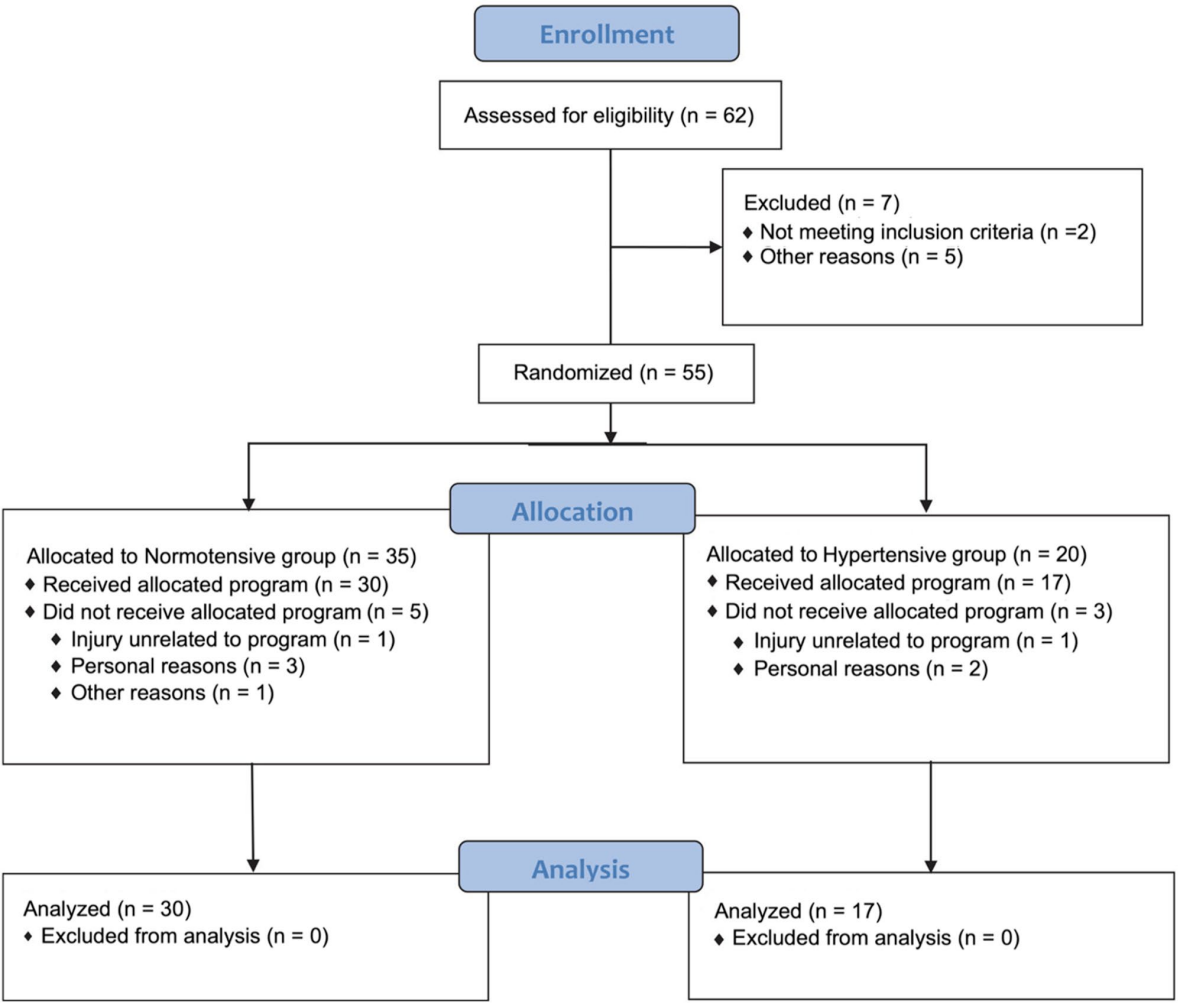
Clinical trial flow diagram

After stratification based on BP levels, participants were randomized to different training sessions using random number generation software, with the allocation sequence concealed. Participants who were unable to complete any part of the prescribed training protocol would have been excluded from the study. The principal investigator (RJ) performed the randomization, and both participants and the statistical analyst (DK) were blinded to group allocation. All participants were fully informed of the study protocol and risks, with written consent obtained. A flow diagram of the study is shown in Fig. 1.

### Procedures

**Fig. 2:**
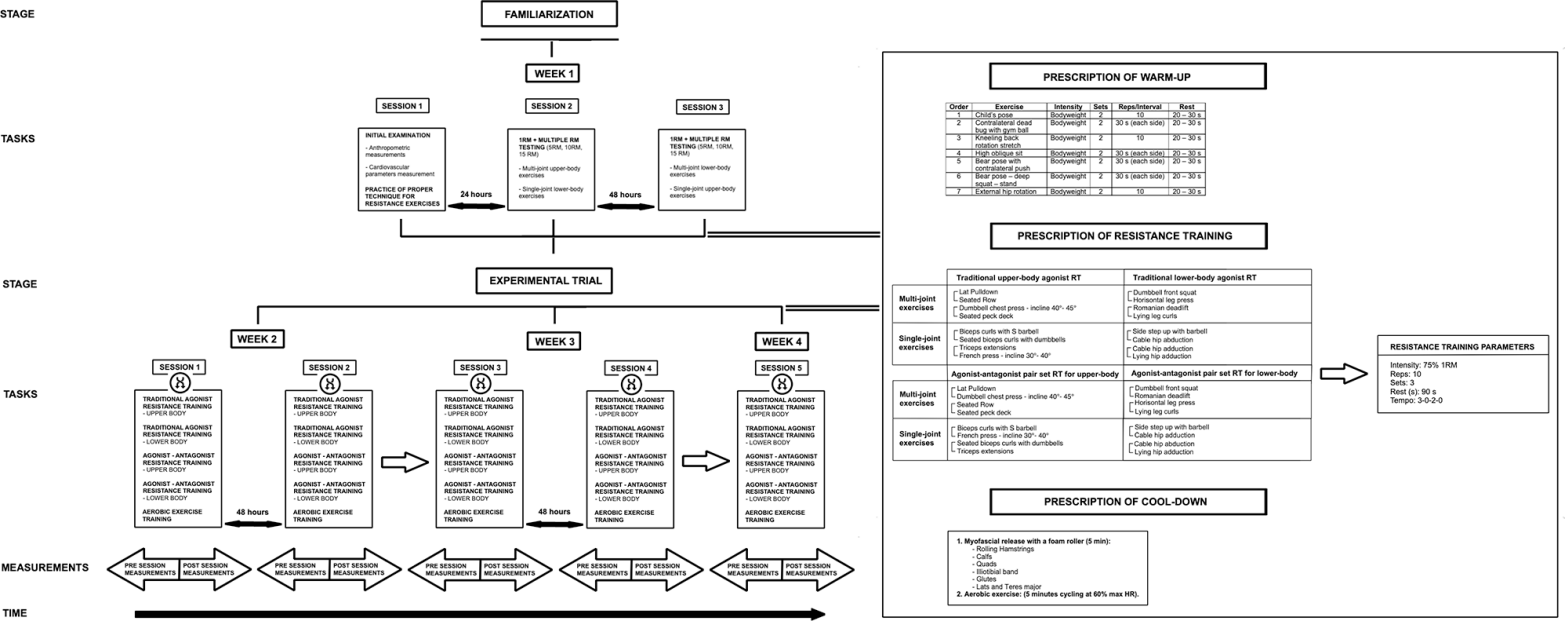
Schematic overview of study design

Sessions followed a counterbalanced order, beginning with anthropometric measurements (height, weight, body composition), then CV assessments (BP and PWVao). These were conducted in a private room within the gym to ensure minimal distractions. Following the initial examination, participants took part in a familiarization session where they practiced the training techniques and performed trial runs before the 1RM and multiple RM testing is shown in Fig. 2.

### 1RM and multiple RM testing

Before 1RM and multiple RM testing, participants completed a standardized warm-up. This included 10 min of cycling at 60% HR max on a Wattbike ergometer, followed by stabilization and mobilization exercises using Dynamic Neuromuscular Stabilization (DNS) (Fig. 2). DNS involves exercises performed in developmental positions aimed at achieving spinal stability and functional joint centration [40]. After the warm-up, strength assessments followed the protocols outlined by Liguori et al. [9], including 1RM and multiple RM tests with 3–5 min rest intervals and a 3-0-2-0 movement tempo. A cool-down phase was added at the end, with a detailed description in Fig. 2. The entire session lasted approximately 90 min. Participants with normotension and stage 1 hypertension performed submaximal loads (15RM, 10RM, 5RM), while only normotensives completed the 1RM testing. All exercises were performed using free weights (Stronggear, Czechia) and cable machines (David Health Solutions, Finland), and participants were familiar with the exercises from their regular training, so no additional instruction was needed.

### Resistance training sessions

Before each RT session, participants performed stabilization and mobilization exercises in the same sequence as during familiarization (Fig. 2). The RT sessions were divided into four methods: (a) AgAnPS RT for lower-body, (b) AgAnPS RT for upper-body, (c) AgPS for upper-body, and (d) AgPS for lower-body. Participants were randomly assigned to each method (Fig. 2). Sessions were held twice a week, with three sets of 10 repetitions at 75% of 1RM per exercise, maintaining strict form. Rest intervals of 90 s were observed, using a 3-0-2-0 tempo. After RT, participants performed myofascial release with a foam roller and light AE (5 min cycling at 60% max HR). All sessions were supervised to ensure proper technique and safety, and conducted in a private gym.

### Aerobic exercises session

One AE session was randomly scheduled alongside RT sessions. The AE session included four 10-minute intervals at 60% max HR, with intensity and duration based on protocols from previous studies [41–43], HR was monitored using a Polar FT4M watch and chest strap (Polar Electro Oy, Finland). This session was performed only once, as this intensity has been shown to be safe for individuals with hypertension [44].

### Cardiovascular parameter assessment

CV parameters were assessed before and during each session, measured three times after each pair of exercises, whether in agonist-antagonist sequence, using the Arteriograph (TensioMed Ltd., Hungary). This device measures vascular stiffness and CV health through a non-invasive suprasystolic occlusion method, detecting pulse waves from the brachial artery with a piezoelectric sensor. Its speed and non-invasiveness are advantages, and its validity is confirmed by invasive oscillometric measurements and central hemodynamic assessments [45, 46]. Data is collected by the device’s software and can be easily exported to MS Excel for analysis. Measurements were taken by a single clinician under standardized conditions, with participants lying supine, following manufacturer guidelines.

### Nutritional and hydratation recommendations

From the familiarization phase, participants followed a standardized dietary program, consuming 3.7 L of fluids daily for men and 2.7 L for women [47], with alcohol strictly avoided. On RT and AE days, large meals, greasy foods, and foods high in saturated or trans fats were to be avoided. On measurement days, high-caffeine and high-sugar foods, as well as salty snacks, were restricted. Participants refrained from consuming coffee, tea, energy drinks, or sweetened caffeinated beverages for at least three hours before measurements [48, 49], and avoided smoking [50] within the hour prior to measurement. They also refrained from eating for two hours before exercise but consumed 500–600 ml of water 2–3 h prior to exercise. During the workout, they were encouraged to drink 180–200 ml of cold water every 15–20 min [51].

### Statistical analysis

Data distribution was assessed using the Shapiro-Wilk test. Descriptive statistics included mean, standard deviation (SD), and standard error (SE) with 95% confidence intervals for normally distributed data, and median with interquartile range (IQR) for non-normally distributed data. To compare sexes and normotensive versus hypertensive individuals, we used Student’s t-test for normally distributed data and Mann-Whitney U test for non-normally distributed data. In case of violated variance homogeneity, the Mann-Whitney U test was applied. One-sample t-tests and Wilcoxon tests compared obtained values with established literature standards. Differences between baseline and exercise values were analyzed with repeated measures ANOVA, with Greenhouse-Geisser correction if needed. Post-hoc Tukey tests were performed for further comparisons. Statistical significance was set at *p* < 0.05. The sample size was calculated a priori using G*Power software (version 3.1, Düsseldorf, Germany) for a repeated measures ANOVA, based on an effect size of d = 0.97, statistical power of 0.80, and significance level of 0.05. These parameters matched those used by Coelho-Júnior et al., [52] in a similar study on CV changes after RT. A sample of 10 participants per group was sufficient. Data analysis was conducted in Microsoft Excel and Jamovi version 1.6.23.0 (The Jamovi Project, United Kingdom).

## Results

A total of 62 individuals were assessed for eligibility, with 55 randomized into two groups: 35 normotensive and 20 hypertensive. Eight participants withdrew (5 from the normotensive group, 3 from the hypertensive group), leaving 30 normotensive and 17 hypertensive participants for the final analysis. The chi-square statistic showed no significant differences in sex-distriution across the groups (X^2^ = 2.65, *p* = 0.10). No adverse effects or significant hypertensive responses were observed. Baseline measures for both groups are shown in Table 1. Both groups had similar RT experience.

Participant adherence to the study protocol remained consistent throughout, with all participants maintaining a minimum of two sessions per week, ensuring a high level of engagement and regularity in the intervention.

**Table 1:** Baseline characteristics of study participants

Charakteristics	Normotensive individuals (*n* = 30)	Hypertensive individuals (*n* = 17)	*p*
Sex (male/female)	12/18	11/6	
Age (years)	47.8 (± 5.9)	54.3 (± 6.0)	0.001
Height (cm)	174.8 (± 10.2)	177.6 (± 11.3)	0.392
Weight (kg)	77.7 (± 15.4)	89.8 (± 16.4)	0.008
Body mass index (kg/m^2^)	25.3 (± 3.6)	28.5 (± 4.6)	0.021
Resistance training experiances (years)	4.2 (± 4.7)	4.6 (± 8.3)	0.247
Systolic blood pressure (mmHg)	120.3 (± 12.0)	135.5 (± 12.0)	< 0.001
Diastolic blood pressure (mmHg)	70.6 (± 8.3)	81.5 (± 7.3)	< 0.001
Aortic pulse wave velocity (ms^-1^)	9.1 (± 2.0)	10.3 (± 1.3)	0.003

Values ale displayed as mean (± standard deviation). Means were compared with independent samples t-test

### Muscle strength test

The results indicated no statistically significant differences in the weight lifted for 1 RM, 5 RM, 10 RM, and 15 RM between men with normal BP and those with first-stage hypertension (*p* > 0.05). Similarly, women with normal BP and those with first-stage hypertension also showed no significant differences (*p* > 0.05). However, a statistically significant difference was identified between the sexes (*p* < 0.05), as detailed in Supplemental file - Muscle strength test results for 15RM, 10RM, 5RM, and 1RM in normotensive individuals and those with stage 1 hypertension.

### The acute changes in systolic blood pressure induced by the different variations of resistance and aerobic exercise training

In individuals with normotension and stage 1 hypertension, no statistically significant differences were found in the changes in SBP when comparing the AgPS, AgAnPS RT, and AE variants. Significant differences (*p* < 0.05) were observed between different body parts such as upper and lower-body, as shown in Table 2. However, no significant differences were found between AgPS and AgAnPS RT for the lower-body and AgPS and AgAnPS RT for the upper-body. Statistically significant differences *p* < 0.05 were found between individuals with normotension and stage 1 hypertension only for superset of exercises AgAnPS RT for lower-body.

In all supersets of AgPS (8.7–24.7 mmHg, SE = 2.0) and AgAnPS RT for the lower-body, a statistically significant increase (AgPS: 8.7–24.7 mmHg, SE = 2.0; AgAnPS RT: 11.3–23.4 mmHg, SE = 2.2; *p* < 0.05) in SBP values were found after the completion of each superset compared to the baseline values in individuals with normotension. In individuals with stage 1 hypertension, significant changes (11.7–24.1 mmHg, SE = 2.6; *p* < 0.05) were also found in all supersets of AgPS for the lower-body. Similarly, significant changes (11.5–23 mmHg, SE = 2.9; *p* < 0.05) were found in AgAnPSRT for the lower-body, with the exception of the 3rd set of Cable Hip Abduction + Lying Hip Adduction. In normotensive individuals, AgPS for the upper-body led to a significant (7.3–10.4 mmHg, SE = 1.9; *p* < 0.05) increase in SBP during Lat Pulldown + Seated Row. In contrast, for Seated Peck Deck + Chest Press, only the 2nd and 3rd sets showed significant (7.3–8.1 mmHg, SE = 1.9; *p* < 0.05) increases. A significant (8.2–10.4 mmHg, SE = 1.9; *p* < 0.05) increase in SBP was found during Biceps Curls with S Barbell + Seated Biceps Curls with Dumbbells. However, all supersets of Triceps Extensions + French Press remained non-significant. In individuals with stage 1 hypertension, every superset of AgPS for the upper body showed significant (11.1–20.8 mmHg, SE = 2.5; *p* < 0.05) increases in SBP. AgAnPS RT for the upper body in normotensive individuals led to significant (7.4 mmHg, SE = 2.0; *p* < 0.05) changes in only one superset, specifically in the 2nd superset of Seated Rows + Seated Peck Deck.

**Table 2:** Comparison of pre- and post-exercise systolic and diastolic blood pressure values across different types of exercise sessions

Type of compared exercise session	Systolic blood pressure			Diastolic blood pressure		
	Mean Difference (mmHg)	SE	*p*	Mean difference (mmHg)	SE	*p*
AgPS lower body vs. AgPS Upper-body	5.2	1.8	0.03	6.4	1.1	< 0.001
AgPS lower body vs. AgAnPS Lower-body	3.2	1.8	0.4	1.0	1.1	0.9
AgPS lower body vs. AgAnPS Upper-body	9.5	1.8	< 0.001	9.7	1.1	< 0.001
AgPS lower body vs. Aerobic exercise	-2.1	1.8	0.8	-2.1	1.1	0.3
AgPS Upper-body vs. AgAnPS Lower-body	-2.0	1.8	0.8	-5.5	1.1	< 0.001
AgPS Upper-body vs. AgAnPS Upper-body	4.2	1.8	0.1	3.3	1.1	0.03
AgPS Upper-body vs. Aerobic exercise	-7.4	1.8	< 0.001	-8.6	1.1	< 0.001
AgAnPS Lower-body vs. AgAnPS Upper-body	6.3	1.8	0.005	8.7	1.1	< 0.001
AgAnPS Lower-body vs. Aerobic exercise	-5.3	1.8	0.03	-3.1	1.1	0.03
AgAnPS Upper-body vs. Aerobic exercise	-11.6	1.8	< 0.001	-11.8	1.1	< 0.001

Values are displayed as mean (± standard error), representing the mean difference (mmHg) in systolic and diastolic blood pressure between two different types of exercise sessions; SE = standard error, p value is from post-hoc Tukey test, with Greenhouse-Geisser correction if needed, AgPS = Agonist paired sets resistance training, AgAnPS = Agonist-antagonist paired sets resistance training

**Fig. 3:**
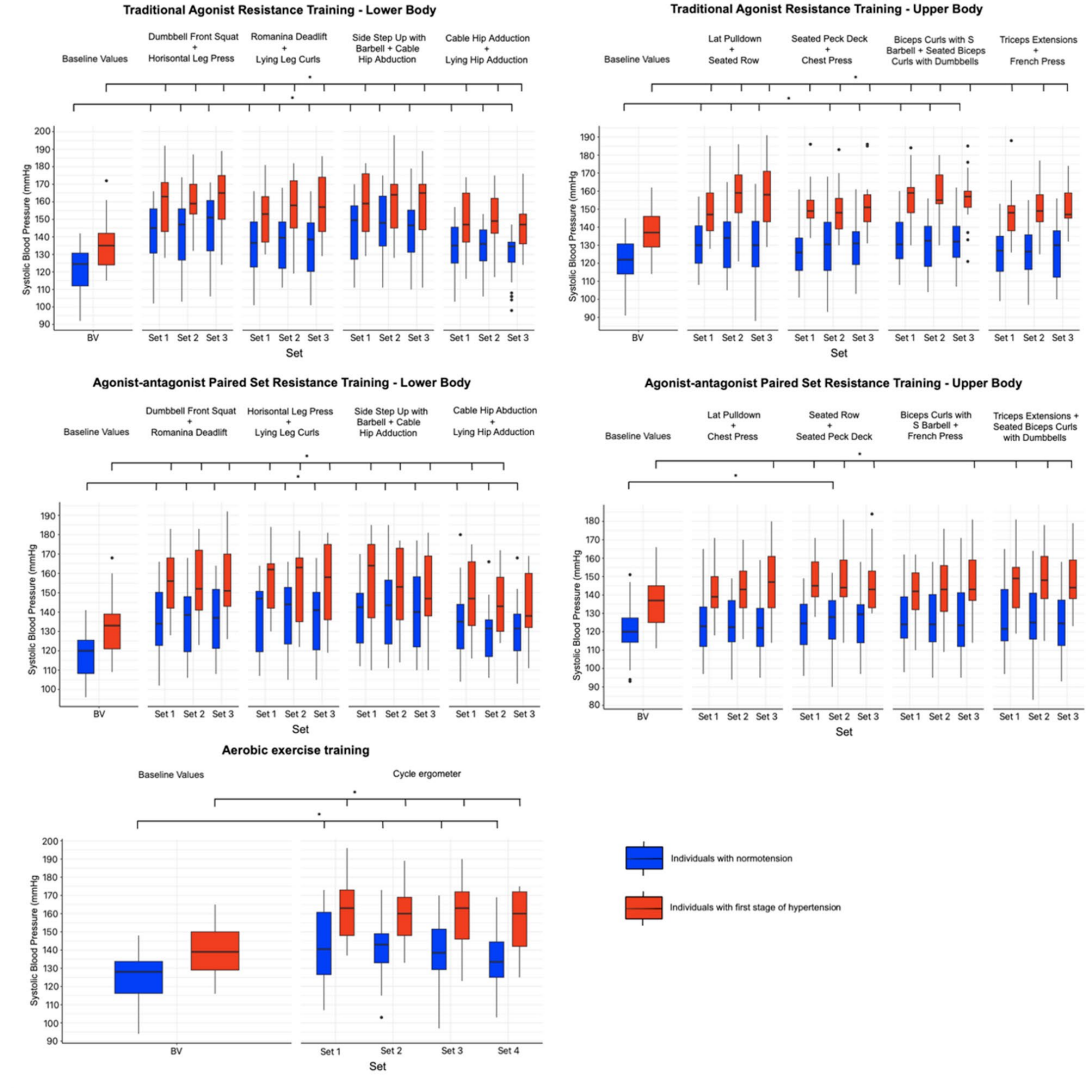
The acute changes in systolic blood pressure induced by the different variations of resistance and aerobic exercise training immediately after the superset of exercises in individuals with normotension and stage 1 hypertension. Values are displayed as median and first and third quartile. *Statistically significant differences between baseline and superset of exercises values, *p* < 0.05. BV = baseline values

In individuals with stage 1 hypertension, significant (10.2–11.1 mmHg, SE = 2.6; *p* < 0.05) increases in SBP were found across all supersets of Seated Rows + Seated Peck Deck and in the 3rd superset of Lat Pulldown + Chest Press. A significant (10.2–12.4 mmHg, 2.6; *p* < 0.05) increase was found in all supersets of Triceps Extensions + Seated Biceps Curls with Dumbbells and only in the 3rd superset of Biceps Curls with S Barbell + French Press (see Fig. 3). In the case of AE, a statistically significant increase in SBP (11.0–18.0 mmHg, SE = 2.3 versus 16.5–23.2 mmHg, SE = 3.1, *p* < 0.05) was found between the baseline values and post-superset values in both individuals with normotension and those with stage 1 hypertension (see in Fig. 3).

### The acute changes in diastolic blood pressure induced by the different variations of resistance and aerobic exercise training

In individuals with normotensio and stage 1 hypertension, no statistically significant differences were found for chages of DBP when comparing the AgPS, AgAnPS RT, and AE sessions. However, there were significant differences (*p* < 0.05) between the types of exercise sessions, as shown in Table 2, with the exception of AgPS and AgAnPS for the lower body, as well as AgPS and AE. Statistically significant differences weren’t found between individuals with normotension and those with stage 1 hypertension across all types of specific exercises for AgPS, AgAnPS, and AE sessions.

**Fig. 4:**
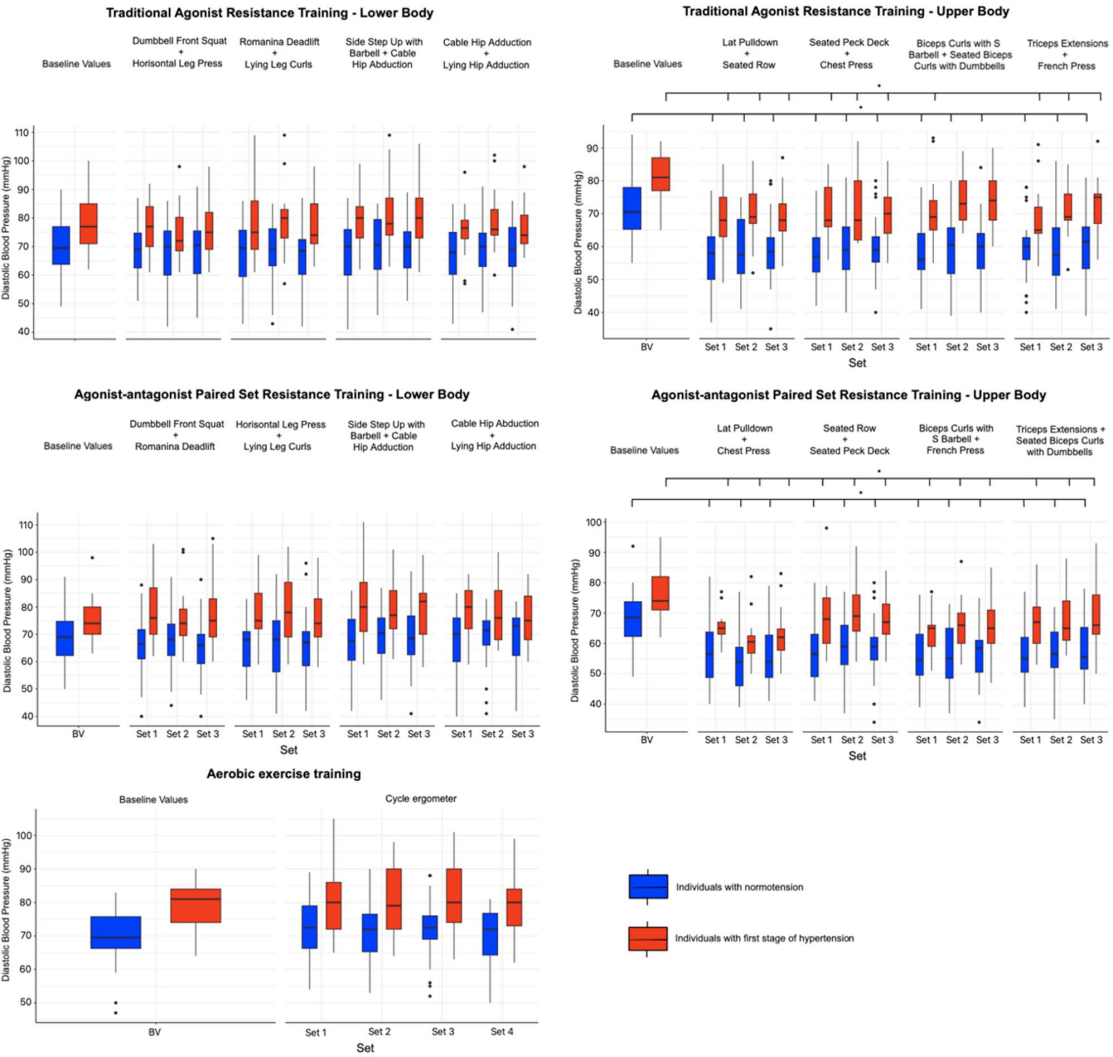
The acute changes in diastolic blood pressure induced by the different variations of resistance and aerobic exercise training immediately after the superset of exercises in individuals with normotension and stage 1 hypertension. Values are displayed as median and first and third quartile. *Statistically significant differences between baseline and superset of exercises values, *p* < 0.05. BV = baseline values

No statistically significant changes in DBP values were found after the completion of each superset of AgPS and AgAnPS RT for lower-body compared to the baseline values in individuals with normotension and stage 1 hypertension. In normotensive individuals, AgPS for the upper-body resulted in a significant (-10.6– -13.7 mmHg, SE = 1.5; *p* < 0.05) decrease in DBP below baseline values across all supersets during the training session. For individuals with stage 1 hypertension, AgPS for the upper body caused a decrease in DBP in the supersets of Lat Pulldown + Seated Row and Seated Peck Deck + Chest Press, as well as in Triceps Extensions + French Press. However, a significant (-8.3– -12.6 mmHg; *p* < 0.05) decrease in DBP was observed only in the 1st superset of Biceps Curls with S Barbell + Seated Biceps Curls with Dumbbells. AgAnPS RT for the upper-body, in both normotensive individuals and those with stage 1 hypertension, led to a significant (-9.2– -13.9 mmHg, SE = 1.3, versus − 6.3– -13.4 mmHg, SE = 1.7; *p* < 0.05) decrease in DBP across all supersets of exercises when comparing baseline and post-superset values.

In the case of DBP, no statistically significant differences were observed between the baseline values and those measured during the AE in individuals with normotension and stage 1 hypertension see Fig. 4.

### The acute changes in aortic pulse wave velocity induced by the different variations of resistance and aerobic training

**Fig. 5:**
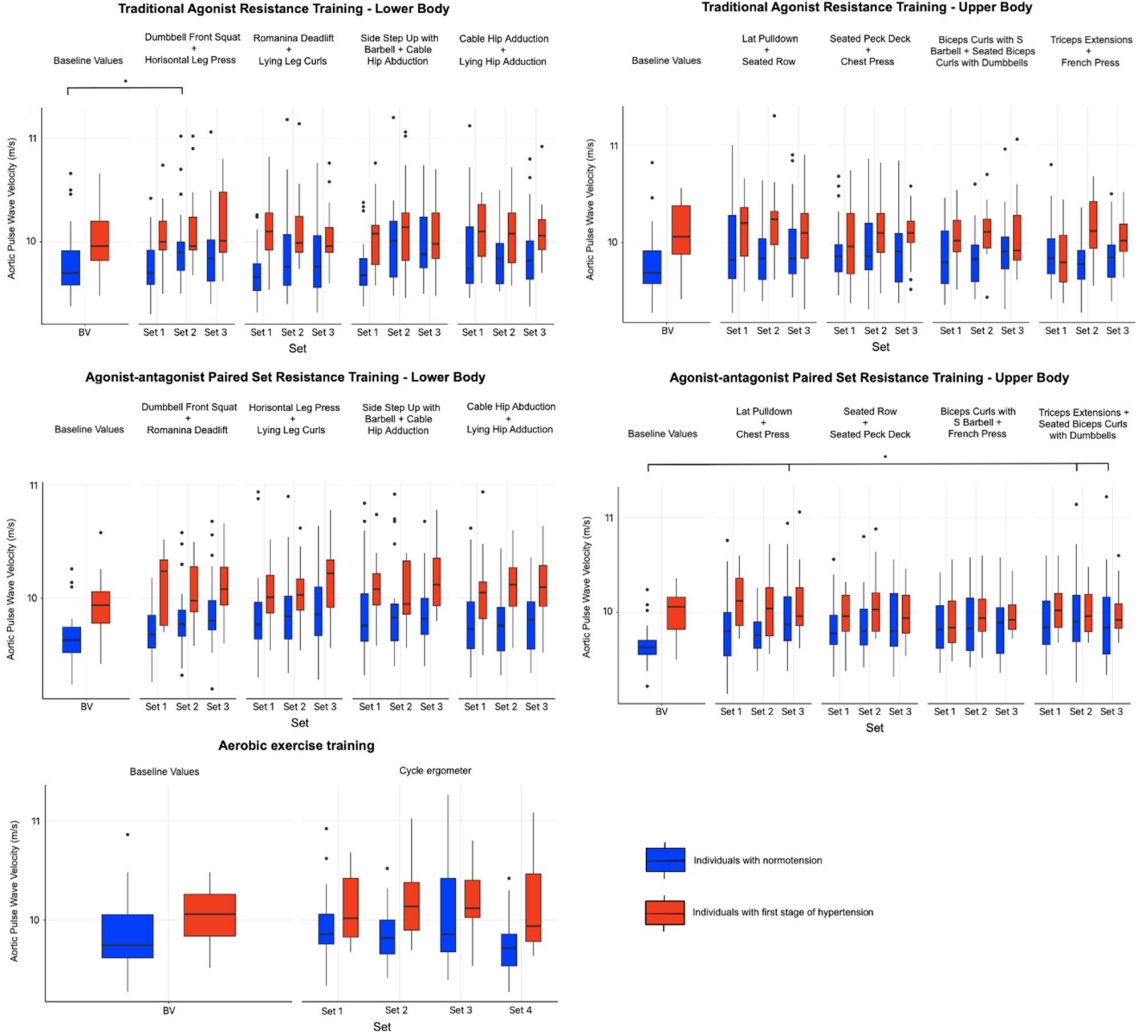
The acute changes in aortic pulse wave velocity induced by the different variations of resistance and aerobic exercise training immediately after the superset of exercises in individuals with normotension and stage 1 hypertension. Values are displayed as median and first and third quartile. *Statistically significant differences between baseline and superset of exercises values, *p* < 0.05. BV = baseline values

In individuals with normotensio and stage 1 hypertension, no statistically significant differences were found when comparing the AgPS, AgAnPS RT, and AE sessions. At the same time, there were no statistically significant differences between AgPS, AgAnPS and AE sessions. Statistically significant differences weren’t found between individuals with normotension and stage 1 hypertension for PWVao across all types of specific exercises for AgPS, AgAnPS, and AE sessions. No significant changes in PWVao values were found after the completion of each supersets of AgAnPS RT for the lower-body compared to the baseline measurements. However, for AgPS targeting the lower-body, a significant change (1.4 ms^-1^, SE = 0.3; *p* < 0.05) was found only in the 2nd superset combining Dumbbell Front Squat + Horizontal Leg Press in normotensive individuals. Several significant changes (1.1–1.2 ms^-1^, SE = 0.3; *p* < 0.05) were found in normotensive individuals during AgAnPS RT for the upper-body, specifically in the 3rd superset of Lat Pulldown + Chest Press and in the 2nd and 3rd supersets of Triceps Extensions + Seated Biceps Curls with Dumbbells. For PWVao, no statistically significant differences were found between the baseline values and those measured during sets of AE see in Fig. 5.

## Discussion

This study explored the effects of AgPS and AgAnPS RT methods on SBP and DBP changes in both normotensive and stage 1 hypertensive individuals with prior RT experience. Our results indicate that both AgPS and AgAnPS RT methods significantly affected SBP and DBP, with similar responses observed between normotensive and hypertensive individuals when identical training parameters were applied.

A key finding was that RT for the lower body, regardless of the method (AgPS or AgAnPS), resulted in a significant increase in SBP, starting from the first set and maintaining this elevated level throughout the exercises. This response was consistent across both groups, suggesting that lower-body exercises, which engage large muscle groups, elicit a more pronounced CV response. These findings align with previous studies, which have shown that lower-body exercises such as squats and deadlifts cause a substantial increase in cardiac output and BP due to venous compression and increased venous return during these movements [53].

Interestingly, no significant difference was observed between AgPS and AgAnPS RT for the lower body in terms of SBP response. However, the intensity and muscle mass involved in these exercises were sufficient to induce a marked increase in SBP, consistent with the known effects of multi-joint exercises, especially when performed at high intensities [13, 54]. These increases are largely attributed to the high CV demand posed by such exercises, where the body must support greater muscle engagement and metabolic cost.

In contrast, exercises targeting the upper body, particularly under AgAnPS RT, did not elicit the same pronounced increase in SBP, especially in normotensive individuals. This suggests that upper-body exercises lead to a more controlled CV response compared to lower-body exercises. Our findings are consistent with those of Blazek et al. [55], who observed that lower-body exercises, particularly those involving large muscle groups, generate higher intrathoracic and intra-abdominal pressures, leading to a more substantial and sustained elevation in SBP. Therefore, upper-body exercises such as the bench press or rows, which exert less pressure on the body, might be better suited for hypertensive individuals or beginners [55].

Regarding DBP, we found that it generally decreased following AgPS and AgAnPS RT for the upper body, particularly in normotensive individuals. In hypertensive individuals, AgAnPS RT also induced a significant decrease in DBP. However, no significant changes in DBP were observed during lower-body exercises or AE. This is consistent with previous research showing that DBP typically drops following a set of resistance exercises and stabilizes shortly afterward [56, 57] The variation in DBP responses underscores the importance of considering individual differences in how the CV system adapts to RT.

Additionally, this study is the first to measure acute changes in PWVao during RT supersets over a 90-minute exercise session. While acute changes in PWVao can be influenced by immediate changes in BP [58]. Significant changes were noted in the normotensive group, particularly during AgPS for the lower body and AgAnPS RT for the upper body. These results support the hypothesis that moderate-intensity RT can lead to an increase in the velocity at which the mechanical wave propagates along the arterial wall in normotensive individuals [59]. However, other studies have reported no significant changes in PWVao after RT at similar intensities [60–63], suggesting that the relationship between exercise intensity, blood pressure, and PWVao may involve additional, complex mechanisms.

In summary, both AgPS and AgAnPS RT methods caused significant increases in SBP, particularly during lower-body exercises. These increases were similar across normotensive and hypertensive individuals, though a slightly greater response was observed in the hypertensive group during AgAnPS RT for the lower body. DBP decreased during upper-body exercises but remained stable during lower-body exercises and AE. The results also indicate that PWVao changes were primarily observed in normotensive individuals. The absence of significant PWVao changes in hypertensive individuals may suggest a differential vascular response, which requires further investigation.

### Strengths and limitations

This study is the first to examine AgAnPS RT in individuals with hypertension, including both normotensive and stage 1 hypertensive participants. The training protocols were carefully structured, with well-defined warm-up, main exercises, and cool-down phases, unlike some previous studies that used random exercises. A major strength of the study is that all measurements were conducted by a single researcher, ensuring consistent adherence to the protocols, including exercise order, intensity, rest intervals, and equipment use. Each session was personalized to the individual, rather than being group-based.

However, the study has some limitations. The results may not be fully generalizable to the broader population, and variations in participants’ body composition, antihypertensive medication use, and existing health conditions could introduce confounding factors.

Future research should include comparisons with untrained individuals with normotension and stage 1 hypertension. While the immediate findings are significant, further studies are needed to explore the long-term effects of different training methods on SBP and DBP.

### Practical considerations

Our findings show that RT significantly increases SBP, particularly during intense exercises. However, changes in SBP, DBP, and PWVao were similar between normotensive and stage 1 hypertensive individuals, except for SBP in lower-body agonist-antagonist exercises. This suggests that both AgPS and AgAnPS RT at 75% 1RM are safe for both groups. Larger muscle mass engagement typically leads to greater pressure increases, while rapid drops in SBP and DBP post-exercise are likely due to muscle vasodilation and baroreceptor responses. Given the variability in CV responses, it’s essential to tailor training protocols such as rest intervals, exercise types, and intensities to individual needs for safety and effectiveness.

## Conclusion

In summary, our study found that both traditional agonist and agonist-antagonist paired set resistance training significantly increased systolic blood pressure during lower-body exercises in individuals with normotension and stage 1 hypertension. Traditional agonist upper-body resistance training also raised SBP, particularly in the first supersets, while agonist-antagonist paired sets had a smaller impact, especially in normotensive individuals. Diastolic blood pressure decreased significantly during upper-body training, but no significant changes were observed for lower-body exercises or aerobic training. Aortic pulse wave velocity remained largely unchanged, except in specific exercises.

Interestingly, no significant differences were found between normotensive and hypertensive individuals. This suggests that moderate-intensity resistance training (75% 1 repetition maximum), whether using traditional agonist or paired exercises, does not cause significant changes in SBP, DBP, or aortic pulse wave velocity across both groups. These findings suggest that both methods are safe and effective for individuals, regardless of blood pressure status.

In conclusion, it’s crucial to tailor training protocols to individual needs to ensure safety and effectiveness, especially given the variability in cardiovascular responses.

## Electronic supplementary material

Below is the link to the electronic supplementary material.

Supplementary Material 1

Supplementary Material 2

Supplementary Material 3

Supplementary Material 4

Supplementary Material 5

Supplementary Material 6

Supplementary Material 7

Supplementary Material 8

Supplementary Material 9

Supplementary Material 10

## Data Availability

Our study adheres to the journal’s data sharing policy. All data supporting our findings are available upon request. For access to the raw data analyzed in this study, please contact the corresponding author, Roman Jurik, at the following email address: roman.jurik@ftvs.cuni.cz.

## Abbreviations

AE: Aerobic exercise
AgAnPS: Agonist-antagonist pair set
ANOVA: Analysis of variance
BP: Blood pressure
CONSORT: Consolidated standards of reporting trials
CV: Cardiovascular
DBP: Diastolic blood pressure
DNS: Dynamic Neuromuscular Stabilization
HR: Heart rate
IQR: Interquartile range
PWVao: Aortic pulse wave velocity
RM: Repetition maximum
RT: Resistance training
SD: Standard deviation
SE: Standard error
SBP: Systolic blood pressure
AgPS: Traditional agonist
