# Supplementary material for: Blood pressure changes during different methods of resistance training in normotensive and stage 1 hypertensive individuals: a repeated measures cross-sectional study
Source: BMC Sports Sci Med Rehabil. 2025 Mar 14;17:49. doi: 10.1186/s13102-025-01097-3 (PMC11907854; doi:10.1186/s13102-025-01097-3)
Supplement: Supplementary file 1 — Supplementary Material 1 [file 13102_2025_1097_MOESM1_ESM.pdf]

Repeated Measures ANOVA - dia

Within Subjects Effects

	Sum of Squares	df	Mean Square	F	p
Time	337.5	1	337.5	13.79	< .001
Time ✻ Intervention	653.2	4	163.3	6.67	< .001
Time ✻ Group	38.2	1	38.2	1.56	0.212
Time ✻ Intervention ✻ Group	278.7	4	69.7	2.85	0.023
Residual	17004.8	695	24.5		

Note. Type 3 Sums of Squares

Between Subjects Effects

	Sum of Squares	df	Mean Square	F	p
Intervention	39246	4	9811.5	54.032	< .001
Group	35053	1	35053.0	193.039	< .001
Intervention ✻ Group	283	4	70.8	0.390	0.816
Residual	126202	695	181.6		

Note. Type 3 Sums of Squares

Post Hoc Tests

Post Hoc Comparisons - Time

Comparison							
Time		Time	Mean Difference	SE	df	t	p_{tukey}
Exercise 3	-	Exercise 4	1.02	0.274	695	3.71	< .001

Post Hoc Comparisons - Intervention

Comparison							
Intervention		Intervention	Mean Difference	SE	df	t	p_{tukey}
AGO-LB	-	AGO-UB	8.384	1.18	695	7.100	< .001
	-	ANT-LB	0.629	1.18	695	0.533	0.984
	-	ANT-UB	12.133	1.18	695	10.275	< .001
	-	CON	-2.179	1.18	695	-1.845	0.349
AGO-UB	-	ANT-LB	-7.755	1.18	695	-6.567	< .001
	-	ANT-UB	3.750	1.18	695	3.175	0.014
	-	CON	-10.563	1.18	695	-8.944	< .001
ANT-LB	-	ANT-UB	11.505	1.18	695	9.742	< .001
	-	CON	-2.808	1.18	695	-2.378	0.123
ANT-UB	-	CON	-14.312	1.18	695	-12.120	< .001

Post Hoc Comparisons - Group

Comparison							
**Group**		**Group**	**Mean Difference**	**SE**	**df**	**t**	**p_{tukey}**
HT	-	NT	10.4	0.747	695	13.9	< .001
