# Supplementary material for: Blood pressure changes during different methods of resistance training in normotensive and stage 1 hypertensive individuals: a repeated measures cross-sectional study
Source: BMC Sports Sci Med Rehabil. 2025 Mar 14;17:49. doi: 10.1186/s13102-025-01097-3 (PMC11907854; doi:10.1186/s13102-025-01097-3)
Supplement: Supplementary file 7 — Supplementary Material 7 [file 13102_2025_1097_MOESM7_ESM.pdf]

Repeated Measures ANOVA - Sys

Within Subjects Effects

	Sum of Squares	df	Mean Square	F	p
Time	9970	1	9969.9	176.83	< .001
Time ✻ Intervention	6649	4	1662.2	29.48	< .001
Time ✻ Group	122	1	122.1	2.17	0.142
Time ✻ Intervention ✻ Group	398	4	99.5	1.76	0.134
Residual	39185	695	56.4		

Note. Type 3 Sums of Squares

Between Subjects Effects

	Sum of Squares	df	Mean Square	F	p
Intervention	22248	4	5562	11.12	< .001
Group	108097	1	108097	216.13	< .001
Intervention ✻ Group	3400	4	850	1.70	0.148
Residual	347606	695	500		

Note. Type 3 Sums of Squares

Post Hoc Tests

Post Hoc Comparisons - Time

Comparison							
Time		Time	Mean Difference	SE	df	t	p_{tukey}
Exercise 3	-	Exercise 4	5.53	0.416	695	13.3	< .001

Post Hoc Comparisons - Intervention

Comparison							
Intervention		Intervention	Mean Difference	SE	df	t	p_{tukey}
AGO-LB	-	AGO-UB	6.140	1.96	695	3.133	0.015
	-	ANT-LB	3.952	1.96	695	2.016	0.259
	-	ANT-UB	10.341	1.96	695	5.276	< .001
	-	CON	-0.970	1.96	695	-0.495	0.988
AGO-UB	-	ANT-LB	-2.189	1.96	695	-1.117	0.798
	-	ANT-UB	4.200	1.96	695	2.143	0.203
	-	CON	-7.110	1.96	695	-3.628	0.003
ANT-LB	-	ANT-UB	6.389	1.96	695	3.260	0.010
	-	CON	-4.921	1.96	695	-2.511	0.089
ANT-UB	-	CON	-11.310	1.96	695	-5.771	< .001

Post Hoc Comparisons - Group

Comparison							
**Group**		**Group**	**Mean Difference**	**SE**	**df**	**t**	**p_{tukey}**
HT	-	NT	18.2	1.24	695	14.7	< .001
