# Supplementary material for: Blood pressure changes during different methods of resistance training in normotensive and stage 1 hypertensive individuals: a repeated measures cross-sectional study
Source: BMC Sports Sci Med Rehabil. 2025 Mar 14;17:49. doi: 10.1186/s13102-025-01097-3 (PMC11907854; doi:10.1186/s13102-025-01097-3)
Supplement: Supplementary file 8 — Supplementary Material 8 [file 13102_2025_1097_MOESM8_ESM.pdf]

Repeated Measures ANOVA

Within Subjects Effects

	Sum of Squares	df	Mean Square	F	p
Time	76.2	4	19.04	9.32	< .001
Time ✻ Intervention	74.2	16	4.64	2.27	0.003
Residual	4844.7	2372	2.04		

Note. Type 3 Sums of Squares

Between Subjects Effects

	Sum of Squares	df	Mean Square	F	p
Intervention	38.0	4	9.50	0.874	0.479
Residual	6447.5	593	10.87		

Note. Type 3 Sums of Squares

Post Hoc Tests

Post Hoc Comparisons - Intervention

Comparison							
Intervention		Intervention	Mean Difference	SE	df	t	p_{tukey}
AGO-LB	-	AGO-UB	-0.01243	0.194	593	-0.0642	1.000
	-	ANT-LB	0.31190	0.198	593	1.5791	0.511
	-	ANT-UB	0.10902	0.192	593	0.5685	0.980
	-	CON	0.11309	0.189	593	0.5984	0.975
AGO-UB	-	ANT-LB	0.32433	0.195	593	1.6629	0.458
	-	ANT-UB	0.12145	0.189	593	0.6418	0.968
	-	CON	0.12551	0.186	593	0.6733	0.962
ANT-LB	-	ANT-UB	-0.20288	0.193	593	-1.0504	0.832
	-	CON	-0.19882	0.190	593	-1.0444	0.835
ANT-UB	-	CON	0.00406	0.184	593	0.0220	1.000
