# Supplementary material for: Blood pressure changes during different methods of resistance training in normotensive and stage 1 hypertensive individuals: a repeated measures cross-sectional study
Source: BMC Sports Sci Med Rehabil. 2025 Mar 14;17:49. doi: 10.1186/s13102-025-01097-3 (PMC11907854; doi:10.1186/s13102-025-01097-3)
Supplement: Supplementary file 10 — Supplementary Material 10 [file 13102_2025_1097_MOESM10_ESM.docx]

Supplementary file - Muscle strength test results for 15RM, 10RM, 5RM, and 1RM in normotensive individuals and those with stage 1 hypertension

| **Muscle strength test results for 15RM, 10RM, 5RM, and 1RM** | | | | | | | | | |
| --- | --- | --- | --- | --- | --- | --- | --- | --- | --- |
| Male | | | | | | **Female** | | | |
| Upper-body resistance exercises | | | | | | | | | |
| Exercises | Individuals | **15RM (kg)** | **10RM (kg)** | **5RM (kg)** | **1RM (kg)** | **15RM (kg)** | **10RM (kg)** | **5RM (kg)** | **1RM (kg)** |
| Lat pulldown | NT | 38.8 (±7.5) | 45.6 (±9.1) | 53.8 (±9.5) | 63.6 (±10.3) | 21.0 (±4.1) | 24.9 (±4.3) | 29.6 (±4.9) | 35.4 (±6.1) |
|  | HT | 37.1 (±6.1) | 44.6 (±8.7) | 51.1 (±9.8) | 57.9 (±11.2) | 19.2 (±4.1) | 23.3 (±3.4) | 28.0 (±2.8) | 34.6 (±3.7) |
| Seated rows | NT | 52.7 (±10.4) | 66.7 (±10.5) | 78.8 (±12.0) | 97.1 (±12.2) | 38.2 (±7.3) | 44.7 (±8.8) | 53.6 (±9.7) | 65.3 (±10.8) |
|  | HT | 59.5 (±11.4) | 69.1(±10.0) | 79.6 (± 11.1) | 86.8 (±25.1) | 34.2 (±3.8) | 41.7 (±5.2) | 50.0 (±7.1) | 60.8 (±10.7) |
| Seated peck deck | NT | 26.0 (±4.2) | 31.9 (±5.7) | 38.3 (±8.4) | 46.5 (±12.5) | 8.9 (±3.0) | 11.5 (±3.3) | 14.2 (±3.7) | 18.1 (±4.9) |
|  | HT | 24.1 (±6.3) | 28.6 (±7.2) | 33.0 (±8.4) | 36.5 (±11.8) | 9.3 (±2.0) | 12.1 (±2.5) | 14.5 (±2.6) | 17.3 (±3.3) |
| Dumbbell chest press – incline 40°- 45° | NT | 24.5 (±8.9) | 28.0 (±9.3) | 38.5 (±11.4) | 44.3 (±11.7) | 9.4 (±2.7) | 13.4 (±2.8) | 18.7 (±4.7) | 23.0 (±5.9) |
|  | HT | 19.6 (±8.9) | 25.8 (±12.0) | 31.1 (±13.3) | 34.0 (±15.1) | 8.0 (±2.8) | 10.8 (±3.0) | 13.9 (±3.8) | 17.3 (±5.2) |
| Biceps curls with S barbell | NT | 17.7 (±4.7) | 22.2 (±5.4 | 27.8 (±6.9 | 35.8 (±9.2) | 8.97 (±2.1) | 11.6 (±2.4) | 14.3 (±3.1) | 17.1 (±4.0) |
|  | HT | 17.5 (±5.5) | 21.9 (±6.3) | 26.6 (±7.5) | 29.2 (±9.1) | 9.3 (±1.3) | 11.7 (±1.9) | 14.4 (±2.9) | 17.8 (±6.1) |
| Seated biceps curls with dumbbells | NT | 16.8 (±3.2) | 22.3 (±4.1) | 27.8 (±5.1) | 34.6 (±8.4) | 7.0 (±2.0) | 9.8 (±2.1) | 13.0 (±2.5) | 17.4 (±4.6) |
|  | HT | 14.4 (±5.2) | 18.1(±6.4) | 22.8 (±7.6) | 27.3 (±8.2) | 6.3 (±2.3) | 8.5 (±2.2) | 10.3 (±2.3) | 13.3 (±2.8) |
| Triceps extensions | NT | 33.1 (±6.0) | 41.3 (±9.0) | 49.2 (±12.8) | 62.5 (±18.0) | 18.6 (±4.8) | 24.0 (±5.2) | 29.2 (±6.2) | 35.6 (±8.0) |
|  | HT | 30.9 (±7.7) | 36.8 (±9.0) | 42.6 (±10.7) | 51.4 (±15.4) | 17.8 (±4.0) | 21.6 (±4.5) | 26.1 (±5.0) | 31.2 (±7.6) |
| French press – incline 30°- 40° | NT | 15.7 (±5.2) | 20.9 (±5.1) | 26.0 (±5.6) | 32.3 (±6.7) | 8.7 (±2.9) | 11.2 (±3.4) | 14.1 (±4.1) | 17.7 (±4.7) |
|  | HT | 16.1 (±3.6) | 20.4 (±3.7) | 25.1 (±4.2) | 29.8 (±4.6) | 10.9 (±1.9) | 13.4 (±2.2) | 16.1 (±3.4) | 19.3 (±5.0) |
| Lower-body resistance exercises | | | | | | | | | |
| Dumbbell front squat | NT | 24.3 (±6.0) | 32.1 (±6.5) | 42.2 (±8.4) | 54.3 (±10.3) | 14.6 (±5.2) | 18.8 (±6.6) | 24.1 (±8.1) | 31.1 (±11.2) |
|  | HT | 21.0 (±4.1) | 26.9 (±6.5) | 33.5 (±8.6) | 41.6 (±13.1) | 13.0 (±2.5) | 17.3 (±4.1) | 21.7 (±4.8) | 27.8 (±6.7) |
| Horizontal leg press | NT | 88.3 (±19.4) | 106.7 (±24.4) | 126.7 (±30.8) | 150.8 (±36.9) | 60.6 (±13.2) | 73.3 (±13.3) | 85.3 (±14.7) | 102.5 (±19.1) |
|  | HT | 93.2 (±17.8) | 108.6 (±20.3) | 127.5 (±24.8) | 148.9 (±27.9) | 58.3 (±10.8) | 67.5 (±14.2) | 80.0 (±16.7) | 93.3 (±23.2) |
| Romanian deadlift | NT | 35.7 (±9.3) | 46.2 (±11.5) | 56.5 (±12.8) | 72.1 (±16.6) | 21.7 (±8.6) | 27.2 (±10.1) | 33.6 (±10.7) | 43.4 (±13.2) |
|  | HT | 32.2 (±14.0) | 37.9 (±12.6) | 44.5 (±14.9) | 53.6 (±17.3) | 18.5 (±5.1) | 23.3 (±7.1) | 28.7 (±9.0) | 35.5 (±12.9) |
| Lying leg curls | NT | 22.9 (±6.2) | 30.0 (±6.7) | 36.9 (±9.1) | 46.5 (±9.2) | 16.5 (±5.5) | 22.2 (±6.5) | 27.5 (±7.3) | 35.6 (±8.0) |
|  | HT | 23.2 (±7.3) | 28.3 (±8.3) | 34.0 (±9.1) | 40.8 (±11.3) | 16.7 (±5.2 | 21.7 (±5.2) | 26.2 (±6.0) | 31.7 (±6.8) |
| Side step up with barbell | NT | 15.2 (±4.7) | 20.4 (±6.7) | 26.5 (±8.7) | 34.8 (±11.6) | 10.1 (±3.9) | 13.6 (±4.3) | 18.2 (±5.9) | 24.6 (±7.2) |
|  | HT | 16.4 (±3.2) | 21.6 (±5.2) | 26.9 (±6.0) | 32.6 (±7.7) | 10.3 (±1.5) | 13.7 (±2.0) | 16.7 (±2.7) | 20.8 (±4.1) |
| Cable hip abduction | NT | 10.8 (±2.0) | 15.8 (±2.0) | 20.8 (±2.0) | 30.0 (±3.0) | 8.4 (±2.6) | 12.9 (±2.5) | 17.8 (±2.6) | 24.7 (±4.0) |
|  | HT | 8.5 (±3.0) | 13.2 (±2.5) | 18.0 (±2.5) | 24.0 (±4.4) | 6.67 (±2.6 | 10.8 (±3.4) | 14.7 (±5.0) | 19.6 (±7.5) |
| Cable hip adduction | NT | 19.6 (±5.4) | 27.1 (±5.8) | 33.8 (±6.4) | 43.8 (±9.3) | 15.0 (±3.8) | 21.1 (±4.7) | 26.7 (±5.4) | 33.1 (±5.7) |
|  | HT | 19.3 (±5.6) | 25.5 (±6.1) | 30.7 (±7.2) | 37.2 (±8.0) | 13.0 (±3.2) | 17.5 (±4.2) | 21.8 (±5.3) | 27.8 (±7.9) |
| Lying hip adduction | NT | 23.8 (±6.1) | 32.1 (±6.2 | 40.8 (±7.0) | 52.1 (±7.8) | 19.7 (±5.0 | 26.1 (±5.6) | 32.1 (±6.0) | 39.5 (±6.3) |
|  | HT | 25.5 (±6.1 | 31.4 (±6.7) | 38.7 (±6.5) | 45.3 (±7.4) | 18.3 (±2.6) | 24.2 (±3.8) | 31.7 (±6.1) | 37.7 (±9.0) |

### Values are displayed as mean (± standard deviation) p <0.05; p<0.05; HT = hypertensive, NT = normotensive, RE = resistance exercise, RM = repetition maximum.
